# High second‐trimester uterine artery impedance correlates to maternal vascular malperfusion and related‐complications

**DOI:** 10.1111/aogs.14486

**Published:** 2022-11-22

**Authors:** Lionel Carbillon, Amélie Benbara, Marion Fermaut

**Affiliations:** ^1^ Department of Obstetrics and Gynecology Hôpital Jean Verdier, Assistance Publique—Hôpitaux de Paris Bondy France; ^2^ Université Sorbonne Paris Nord Bobigny Île‐de‐France France

Sir,

Stevens et al.^1^ recently concluded in a retrospective analysis of data from a prospective cohort of pregnancies with clinical phenotypes of placental syndrome (eg pre‐eclampsia and/or fetal growth restriction [FGR]), that “decidual vasculopathy”, one of the features defining maternal vascular malperfusion (MVM), was associated with a pulsatility index above the 95th centile in uterine artery Doppler (UtA‐PI) “independent of clinical phenotype”.

We believe that several points in the rationale and design of this study^1^ raise important questions challenging the conclusions. First, in the study design, FGR was wrongly only defined as a small‐for‐gestational‐age fetus (estimated fetal weight <10th centile or abdominal circumference <10th centile). However, a Delphi consensus process has recently been widely accepted in the Netherlands^2^ and worldwide, ruling that estimated fetal weight/abdominal circumference below the 10th centile is not sufficient, a PI above the 95th centile in the umbilical artery or UtA must be included in the definition. Second, the authors recognized the potential misclassification of fetuses with limited growth due to non‐placental causes among the cases, but they also overlooked the possible misclassification of FGR cases with “late‐normalization” of PI, because they measured UtA‐PI “as close as possible to delivery”, and did not take into account the progressive decrease in the mean UtA‐PI until the late stages of pregnancy.^3^ Birthweight has long been correlated with the timing of UtA‐PI “normalization” early from 19–21 weeks of gestation or a little later at 24–26 weeks,^4^ depending on the quality of the placentation underlying this process.^4^

In this regard, as intrauterine fetal death is a common complication of unrecognized FGR/MVM, Amodeo et al.^5^ have assessed the relations between second‐trimester UtA‐PI and histologic lesions of MVM after stillbirth, vs matched controls with pre‐eclampsia and/or FGR having a live birth. These authors stratified for UtA‐PI within the same multiples of the median quartile UtA‐PI values in the two groups. FGR was defined according to the international Delphi consensus.^2^ Importantly, the rates of FGR and pre‐eclampsia increased with increasing UtA‐PI multiples of the median quartiles, and Amodeo et al.^5^ found that in the two highest UtA‐PI quartile values, the proportion of FGR was considerably high in both the stillbirth and control groups (68% and 92%, respectively). Furthermore, retroplacental hematoma and distal villous hypoplasia were significantly more common in the stillbirth group. In contrast, although decidual vasculopathy and placental infarction were significantly more common in the stillbirth group, these differences were not statistically significant. Especially, logistic regression showed, with growing quartiles of UtA‐PI, increasing rates for MVM (retroplacental hematoma, placental infarction, distal villous hypoplasia, accelerated villous maturation); placental infarction and distal villous hypoplasia were the MVM features more highly associated with the increasing quartile UtA‐PI measurements.

Routine second‐trimester UtA Doppler appears justified and the finding of abnormal UtA velocimetry supports close later monitoring targeting FGR and early‐onset pre‐eclampsia screening, which could help to refine with accuracy the best timing for possible betamethasone/magnesium treatment and optimized elective delivery, especially in the prevention of stillbirth.
